# 2217: A transgenic retinitis pigmentosa zebrafish model for drug discovery

**DOI:** 10.1017/cts.2017.30

**Published:** 2018-05-10

**Authors:** Logan Ganzen, Chi Pui Pang, Mingzhi Zhang, Motokazu Tsujikawa, Yuk Fai Leung

## Abstract

OBJECTIVES/SPECIFIC AIMS: Retinitis pigmentosa (RP) is a hereditary retinal degeneration disease that affects ~1 in 4000 individuals globally, and there are currently no effective treatment options available. In order to identify potential drug treatments, we optimized our existing a behavioral assay around a transgenic zebrafish carrying a truncated human rhodopsin transgene [Tg(rho:Hsa.RH1_Q344X)]. This line was also crossed with the Tg(-3.7rho:EGFP) reporter for rod visualization. The Q344X larvae experiences significant rod photoreceptor death by 7 days postfertilization (dpf) (Nakao *et al*., 2012). METHODS/STUDY POPULATION: To assess the vision of the Q344X zebrafish, the VMR assay was run under a dim-light condition based on recorded rod b-waves in larval fish (Moyano *et al*., 2013) and the minimum cone activation threshold in mice (Cachafeiro *et al*., 2010). Specifically, Q344X and control larvae at 7 dpf were placed into a 96-well plate and acclimated to a dim-light source (1.802e-05 μ W/cm^2^ at 500 nm) for 1 hour. The VMR was tracked and quantified during light offset. The total distance traveled was averaged and analyzed at 1 second poststimulus. Retinas were dissected from Q344X and control larvae and whole-mounted to validate the rod degeneration in the Q344X model. RESULTS/ANTICIPATED RESULTS: We found that the Q344X larvae displayed an attenuated VMR (0.121±0.041 cm) to the dim-light offset as compared with the control larvae (0.2751±0.038 cm) (two-sample *t*-test; *p*-value=4.619e-14, n=19). Analysis of whole-mounted retinae indicated significant rod degeneration at 7 dpf compared with controls (control: 87 rods/retina, Q344X: 9.3 rods/retina, Welch two-sample *t*-test, *p*-value=1.4e4). It is unlikely that the cones of the zebrafish contributed to this VMR since the light intensity of the assay was below the cone detection threshold of mice. As the only apparent difference between the 2 groups of larvae is significant rod degeneration, it can be concluded that the behavioral phenotype was a result of the degeneration. DISCUSSION/SIGNIFICANCE OF IMPACT: These results suggest that the attenuated Q344X VMR is a result of the rod photoreceptor death. This behavioral phenotype can be taken advantage of to develop a drug screening assay. Future steps will screen chemical libraries to identify compounds that ameliorate the rod degeneration. Compounds that prevent degeneration are expected to result in a significant increase in locomotion in response to the dim visual stimulus.

